# Quality of inpatient care of small and sick newborns in Pakistan: perceptions of key stakeholders

**DOI:** 10.1186/s12887-021-02850-6

**Published:** 2021-09-10

**Authors:** Nousheen Akber Pradhan, Ammarah Ali, Sana Roujani, Sumera Aziz Ali, Samia Rizwan, Sarah Saleem, Sameen Siddiqi

**Affiliations:** 1 Department of Community Health Sciences, Aga Khan University, Karachi, Pakistan; 2 Department of Epidemiology, Columbia University, New York City, NY USA; 3 United Nations International for Children’s Education Fund, Country Office, Islamabad, Pakistan

## Abstract

**Background:**

In LMICs including Pakistan, neonatal health and survival is a critical challenge, and therefore improving the quality of facility-based newborn care services is instrumental in averting newborn mortality. This paper presents the perceptions of the key stakeholders in the public sector to explore factors influencing the care of small and sick newborns and young infants in inpatient care settings across Pakistan.

**Methods:**

This exploratory study was part of a larger study assessing the situation of newborn and young infant in-patient care provided across all four provinces and administrative regions of Pakistan. We conducted 43 interviews. Thirty interviews were conducted with the public sector health care providers involved in newborn and young infant care and 13 interviews were carried out with health planners and managers working at the provincial level. A semi-structured interview guide was used to explore participants’ perspectives on enablers and barriers to the quality of care provided to small and sick newborns at the facility level. The interviews were manually analyzed using thematic content analysis.

**Findings:**

The study respondents identified multiple barriers contributing to the poor quality of small and sick newborn care at inpatient care settings. This includes an absence of neonatal care standards, inadequate infrastructure and equipment for the care of small and sick newborns, deficient workforce for neonatal case management, inadequate thermal care management for newborns, inadequate referral system, absence of multidisciplinary approach in neonatal case management and need to institute strong monitoring system to prevent neonatal deaths and stillbirths. The only potential enabling factor was the improved federal and provincial oversight for reproductive, maternal, and newborn care.

**Conclusion:**

This qualitative study was insightful in identifying the challenges that influence the quality of inpatient care for small and sick newborns and the resources needed to fix these. There is a need to equip Sick Newborn Care Units with needed supplies, equipment and medicines, deployment of specialist staff, strengthening of in-service training and staff supervision, liaison with the neonatal experts in customizing neonatal care guidelines for inpatient care settings and to inculcate the culture for inter-disciplinary team meetings at inpatient care settings across the country.

## Introduction

Facility-based newborn care refers to the specialized health care services provided by skilled health care personnel 24/7 at the secondary and tertiary care units [1, 2]. Improving the quality of inpatient care is instrumental in reducing neonatal mortality rate (NMR), particularly in low and middle-income countries (LMICs); which accounts for 98% of neonatal deaths [3, 4]. In pursuit of achieving the target of reducing neonatal mortality to 12 per 1000 live births of Sustainable Development Goal (SDG) 3 [5], it is imperative to improve the quality of inpatient care services. Also, stillbirths account for over half of the perinatal deaths, are often underreported, and remain an unprioritized agenda across the globe [6]. Despite slower progress in reducing neonatal mortality globally; facility-based neonatal care has remained under-prioritized and underfunded, particularly in resource-constrained settings [7].

Globally, Pakistan has been reported to have the highest NMR and the country has been regarded as the riskiest place for a child to be born [8]. Federal and provincial departments of health have addressed neonatal care in Maternal, Newborn, and Child Health (MNCH) plans [9]. Also, United Nations International for Children’s Education Fund (UNICEF) has supported the Government of Pakistan in establishing 38 Sick Newborn Care Units (SNCUs) at public health care facilities in the country [10]. Collaborative efforts have helped in declining NMR in the country from 55 deaths per 1000 live births to 42 deaths per 1000 live births during the last five years [11]. However, the number of newborn deaths is still unacceptably high.

While designing effective strategies to improve the quality of inpatient care for newborns, it is imperative to understand context-specific factors. Therefore, the closure of quality gaps in inpatient care services for small and sick newborns, among others, demands identifying the health system bottlenecks from stakeholders’ (frontline health care delivery staff, planners, and implementers’ at the provincial level) perspectives to address the gaps in neonatal inpatients care. Strengthening of the existing facility-based system for the care of sick children is the most practical and cost-effective approach to avert preventable newborn deaths and is also central to achieving the goals of the Every Newborn Action Plan (ENAP) [12]. To date, newborn survival in Pakistan has received attention through various research and policy interventions. The research in this area is mainly clustered around establishing step down units at the facility for low birth weight babies [13], economic evaluations to measure maternal and neonatal health (MNH) service costs [14], assessment of service readiness in offering MNH services at facility level [15], assessing the outcomes for neonatal care admissions [16] to health workers’ competency assessment on MNH aspects in the country [17]. Besides this, integration of neonatal health into the existing national and provincial MNCH program [18], and inclusion of neonatal component in integrated management of neonatal and childhood illnesses strategy, since 2000 are some of the policy level initiatives [19].

Exploring perspectives of the frontline care providers of neonatal care and health sector planners and managers has received less attention in unfolding the bottlenecks at the health systems level. This paper, therefore, presents findings from the qualitative study with the key stakeholders to explore their perceptions regarding the factors influencing the quality of care for small and sick newborns at inpatient care facilities in Pakistan.

## Methods

### Study design

This qualitative exploratory study is part of a larger cross-sectional study to assess the readiness and quality of inpatient care for NYIs provided in public sector hospitals across Pakistan [20]. The larger study utilized the mixed methods approach. The findings from the qualitative arm of the study related to stakeholder’s perspectives for the quality of inpatient care for small and sick newborns at inpatient care facilities are presented in this paper.

### Study sites

The data for the study was gathered from a sample of 23 public sector hospitals spread across all the provinces and regions of the country, providing inpatient care for newborns and young children (0–59 days of age). Of the 23 public sector health care facilities, 12 facilities were supported by UNICEF (in terms of infrastructure for sick newborn care), while the remaining 11 facilities were without UNICEF support. All facilities were purposively selected in consultation with the UNICEF country office, and the Ministry of National Health Services Regulation & Coordination. These included 3 tertiary care hospitals, 6 provincial/regional referral hospitals, and 14 District Headquarter (DHQ) Hospitals. And, offices of the Director-General (DG) Health across four provinces and two federal territories.

### Study participants and sampling criteria

Using purposive sampling approach, key informant interviews were conducted with the two groups of respondents. The first group includes healthcare providers (*n* = 30) directly or indirectly involved in the care of newborns and young infants including in-charge of health facilities (Medical Superintendent-MS) or their representatives, and service managers with responsibilities related to newborns and young infants (NYIs) care. This group includes paediatricians, neonatologists, obstetricians, nurses, gynecologists, and medical superintendents with age range of 37–57 years and varying years of experience (10–16 years) at public sector hospitals. Up to five respondents per facility (inclusive of the medical and nursing workforce) were interviewed. Where there was a choice, the most experienced person in providing the neonatal care service, present at the time of the assessment was interviewed. The second group include managers and planners (*n* = 13) at the district and provincial level including DG Health Services, MNCH program Director, and MNCH Program Managers at the Department of Health in all four provinces of the country. The participants were in age range of 44–57 years. Most of them had MBBS degree with post-graduation in public health and health care management, with experience of approximately 7–15 years. The average duration of interviews was 45–60 min.

### Data collection method

In-depth interviews were conducted using a semi-structured interview guide to study barriers and enablers for inpatient care of small and sick newborns in Pakistan (Table 1). All study participants were interviewed after explaining the purpose of the study and obtaining written informed consent. The questions focused on the quality of inpatient care for small and sick newborns at the respective facilities, availability of infrastructure support, availability and expertise of specialized staff, referral system, and training of staff in neonatal care. Special emphasis was given to the associated challenges in the existing health care system in the provision of neonatal care at the facilities. This paper was not looking at the strategies to address implementation issues related to NYI care in inpatient care settings. The solutions to implementation bottlenecks are addressed in the main paper.

**Table 1:** In-depth interview guide to explore the quality of inpatient care for small and sick newborns in Pakistan

S. #	Questions with probes
**1**	What are your views on the status of child health (particularly facility based care for small and sick newborns) in Pakistan? ▪ Progress made so far ▪ Urban vs. rural settings ▪ Quality of care aspects
**2**	Based on your experience, how do you perceive the quality of care for small and sick newborns at inpatient care units of your hospital? ▪ Availability of standards/ guidelines ▪ Infrastructure support ▪ Care practices for sick newborn ▪ Monitoring and supervision of staff ▪ Any other (if any)
**3**	Please comment on the availability of trained staff for small and sick newborn care at inpatient care units of your hospital. ▪ Adequate or inadequate availability of specialist cadres ▪ Availability of trained staff ▪ Capacity building needs ▪ Staff turnover ▪ Any other (if any)
**4**	Comment on the existing referral system for referring sick newborns from your facility to the next level. ▪ Issues experiences/ observed ▪ Areas needing improvement/recommendations ▪ Any other
**5**	Overall, which factors do you consider that act as a barrier in provision of quality care to small and sick newborns at the health facility level? ▪ Organizational support ▪ Inadequacies in the infrastructure ▪ Monitoring and supervision practices ▪ Staff availability and required competencies ▪ Neonatal case management ▪ Any other
**6**	What factors do you consider having a potential to improve/support the quality care to small and sick newborns at the health facility level? ▪ Any initiatives (if any) taken at the hospital level ▪ Any initiatives (if any) at the district health department ▪ Any initiatives (if any) at the provincial health department ▪ Any other

This table presents the interview guide with questions and probes that were referred during interviews with the study participants

All the interviews were held during April–May 2019 and were conducted bilingually i.e., Urdu and English language.

### Analysis

The data gathered through interviews in the local language was transcribed and translated into English. Transcripts were proofread multiple times by the lead author (NP), and two co-authors (AA and SaR) to get the correct understanding of the information. For this purpose, we were guided by the work of Mcellan et al [21] and of Bailey [22]. The transcripts were manually analyzed using Graneheim & Landman, 2004 content analysis approach [23]. The main ideas on the transcripts were selected and labeled as “codes”. Similar codes were categorized and assembled into categories. In the final step, sub-categories with similar concepts were grouped into “themes” (Table 2). Meetings were held among the team members to mutually agree on the codes, categories, and themes derived from the qualitative data set. The confidentiality of the participants was maintained by assigning a unique code to safeguard their identity. The purpose of our analysis was not to draw comparisons regarding the perceptions of providers from UNICEF-supported and non-UNICEF supported public health care facilities. It was rather to highlight their perceptions regarding the factors influencing the quality of care for small and sick newborns at inpatient care facilities in Pakistan.

**Table 2:** Findings from the in-depth interviews of the key stakeholders

Theme I: Barriers contributing to the poor quality of inpatient care for small and sick newborns across Pakistan		
S.#	Categories	Codes
1.	Lack of essential neonatal care standards	▪ Protocol should be available ▪ Unavailability of newborn care guidelines ▪ Develop proper guidelines ▪ One child per bed ▪ System of protocols ▪ Criteria
2.	Inadequate infrastructure and equipment for the care of small and sick newborns	▪ No electricity ▪ Unavailability of NICUs ▪ Unavailability of labor rooms ▪ Unavailability of space ▪ Absence of baby cots ▪ Operation theater is non-functional ▪ Ultrasound facility ▪ Early discharge of preterm ▪ No ambulatory bags ▪ No Incubators
3.	Issues with neonatal health care workforce	
3.1.	Deficiency of specialized workforce for neonatal case management	▪ Shortage of manpower ▪ Inadequate staff ▪ Single neonatologist ▪ Cannot find paediatricians ▪ Neonatologist not available ▪ One nurse in evening and night shift ▪ Staff shortage ▪ Golden minutes wasted ▪ Child specialist on call
3.2	Inadequate thermal care management for newborns	▪ Child still cold ▪ Hypothermia ▪ Newborn dying ▪ Baby handed over to TBAs ▪ Lack of thermal care ▪ TBAs do not keep the baby warm
4.	Inadequate referral system	▪ No proper referral or support ▪ Must send him (patient) forward ▪ Unavailability of transportation ▪ No follow up after referral ▪ Delay at DHQ level ▪ Effective communication in emergency situations
5.	Absence of a multidisciplinary approach in neonatal case management	▪ Neonatologist do not reach on time ▪ Communication gap ▪ Lack of coordination ▪ Need for teamwork
6.	Need to institute strong monitoring system to prevent neonatal deaths and stillbirths	▪ Review of reports ▪ 10 deaths in one month ▪ Verbal autopsies ▪ Cause of stillbirths and neonatal deaths ▪ Audits and follow ups ▪ Training needs ▪ No audits
**Theme II: Enablers contributing to improved inpatient care for small sick newborns**		
1.	Improved federal and provincial oversight for reproductive, maternal, and newborn (RMNCH) care	▪ Technical group ▪ Actively working ▪ WHO and UNICEF ▪ INGOs ▪ Promoting RMNCH care ▪ Evidenced based interventions ▪ Umbilical cord care

This table presents the themes, categories, and codes arriving from the in-depth interviews of the key stakeholders to assess their perspectives of the quality of in-patient care for small and sick newborns across public sector health care facilities in Pakistan

### Findings

The major findings obtained from participant’s perceptions are grouped under two broad themes; (1) Barriers contributing to the poor quality of inpatient care for small and sick newborn care. This includes perceived bottlenecks and challenges impeding the quality of small and sick newborn care, and (2) Enablers contributing to improved inpatient care for small and sick newborns. This theme identified one factor potentially contributing to improved care for small and sick newborns. These two themes are explained in the following sections.

### Theme I: barriers contributing to the poor quality of inpatient care for small and sick newborns across Pakistan

#### Category 1: lack of essential neonatal care standards

Both groups of respondents (health care providers and planners) expressed their dissatisfaction over the quality of care provided to small and sick newborns at the district and tertiary care units across Pakistan. Among the underlying reasons, the non-availability of essential newborn care guidelines/standards at the facility by frontline service delivery staff were highlighted as the major concern. A group of paediatricians and senior representatives at the MNCH Provincial Directorate voiced their concerns by highlighting the non-availability of standards protocols prevent intrapartum stillbirths and neonatal deaths and common childhood illnesses and recommended for the development of these standards at the provincial and district level.

“There are no protocols. No standard protocols are being followed for newborn and neonatal care.” (MNCH Assistant Director, 005–01).

“They (district and the provincial department of health officials) should make proper guidelines for the management of major issues such as birth asphyxia, preterm infections, etc.” (Paediatrician, 003–05).

A respondent while reflecting the current practice of keeping 3–4 neonates in a single cot, stated;

“Now high standard means one child per bed. In that room (nursery), there are four newborns in one bed. So, what kind of standard we are fulfilling?” (Gynecologist, 001–02).

An obstetrician expressed concerns that the absence of set guidelines encouraged malpractices related to the care of a newborn;

“There is no newborn care as such. An obstetrician will try to save the woman during delivery and will hand over the baby to traditional birth attendant (TBA) (Dai/ Aya), and they will turn the baby upside down and slap her to make her cry. No vitals, no APGAR score, no explanation regarding the danger signs is carried out. Even at the tertiary level, the system is not up to the mark. So, what can you expect from Rural Health Center, Basic Health Units, and DHQs?” (MS, 001–03).

While respondents shared their grievances about the absence of neonatal care standards, they also acknowledged World Health Organization (WHO) protocols of essential maternal and neonatal care and highlighted the need for implementing the same at SNCUs.

#### Category 2: inadequate infrastructure and equipment for the care of small and sick newborns

Health care providers including the MS, obstetricians/ gynecologists, and pediatricians also expressed concerns about the inadequacy of infrastructure and equipment in health facilities (belonging from non-UNICEF supported facilities); as an important reason for the poor quality of neonatal care. Infrastructural deficiencies included unavailability or non-functionality of neonatal intensive care units (NICU), labor room, ventilators, life-saving medications, shortage of the required number of infant cots and ultrasound facility, non-functional operation theaters, etc. The resulting poor quality of care contributed to neonatal deaths at these facilities.

“There are a lot of neonatal deaths in the facility as well. The reasons are that we have a shortage of manpower, the need for electricity is not fulfilled. … …… If the incubator is required, it’s not provided … … ..there are few medicines … ..these are the problems due to which neonatal deaths are reported”. (Obstetrician, 001–04).

“A preterm baby, less than 28 weeks needs ventilator support, which we are unable to provide. So, what do you expect? How can we make progress in this situation?” (Gynecologist, 002–03).

Frontline care providers believed that an early discharge of preterm neonates is due to limited bed capacity at the DHQ hospitals.

“One issue is the unavailability of space, because of which at times we are compelled to discharge preterm neonates” (Paediatrician, 002–03).

Furthermore, the paediatricians also shared their views about the compromised quality of care in the absence of required logistics and essential lifesaving medications for the sick newborns;

“..........If a child is presented with fits and I don’t have valium or when a newborn is not breathing, I don’t have an ambu bag to resuscitate, the baby will die. This equipment should be made available”. (Paediatrician, 007–09).

Furthermore, both groups of respondents also highlighted the scarcity of incubators and ventilators in rural areas. Citing this a stakeholder at the provincial level (under which both UNICEF and non-UNICEF supported facilities fall), stated;

“After delivery, if the baby gets hypothermia, fever or asphyxia then there is no neonatal set-up in the rural areas to manage such cases. Our ambulances are not equipped with ventilator services. They do not have the incubator or the warmer …” (MNCH Assistant Director, 005–01).

Moreover, some of the respondents at the facility level also shared their grievances about the inadequacy of oxygen supply and related equipment (ambulatory bags, oxygen flow meters, etc.).

#### Category 3: issues with neonatal health care workforce

##### Sub-category 3.1: deficiency of specialized workforce for neonatal case management

Inadequate availability of the specialized workforce for neonatal case management was perceived as another hurdle in delivering optimum quality of care. Both groups of respondents equally identified the shortage of neonatologists, pediatricians, and nurses in the neonatal units.

A pediatrician while highlighting the dearth of required workforce exclaimed;

“… …. We do not have a single neonatologist in the entire province. Paediatricians deal with neonates. They are not present in every DHQ. The staff is not enough across different units of the hospital. …” (Paediatrician, 007–04).

Respondents while reflecting the unavailability of a specialized workforce round the clock at the inpatient care units, shared that evening and night shifts are mostly observed without nurses. Alongside the unavailability of nurses, paediatricians were reported to be on call and were only reported to be accessible in the daytime; resulting in the delay of essential newborn care.

“The patient care is affected due to the shortage of staff in night shifts. Let’s suppose cesarean has been done and now they need a child specialist, but the child specialist is on call …. During this calling process, the golden minutes are wasted. And when the pediatrician arrives, the baby has died already, or he has been resuscitated.” (MNCH Assistant Director, 005–01).

Dialogues with the MNCH program planners and managers unfolded the reasons contributing to the dearth of the specialized workforce at inpatient care units. A commonly reported reason was the budgetary constraints at the provincial level; restricting recruitment of skilled workforce. While highlighting the inadequacy of the specialized neonatal care workforce across DHQs, suggestions were proposed for in-service training of residents in neonatology.

“There should be more people sent for the capacity building. Those who have done house jobs in paediatrics should be sent to neonatology units. They should learn and build their capacity and then get appointed in the districts.” (DG Health, 007–02).

##### Sub-category: 3.2. Inadequate thermal care management for newborns

While respondents highlighted multiple aspects of poor quality of newborn care at the facility level, it came under discussion from one province that newborns are handled by the TBAs; resulting in inadequate thermal care, which leads to neonatal deaths at the facility.

“… … .. So, what I have observed is that the child is handed over to the TBA, she takes the child somewhere and doesn’t keep him warm …. They just dry the child with a cloth and the child remains cold later. A lot of children die because they remain cold.” (Paediatrician,001–01).

One of the senior officials at the Provincial Department of Health also shared similar malpractice after childbirth.

“Soon after the delivery, if the male baby is born, he is handed over to ward servants who then hand over him to family. They wander all-around taking the baby thus causing hypothermia. This has been strictly mentioned a lot of times that baby should be placed in front of the heater or the baby warmer should be switched on at least half an hour before the delivery.”

(DG Health, 001–01)

#### Category 4: inadequate referral system

Across the regions, the inadequate referral system in general and in particular to neonatal cases was repeatedly emphasized. While unfolding the issues in the referral system, participants reported a lack of pre-referral care and treatment by attending physicians reflecting the incompetency of frontline staff in managing emergency cases requiring urgent referrals.

“… .. The doctors are incapable of resuscitation and proper referrals, this leads to a situation where a critically ill patient arrives with no proper referral or support, so we must send him forward.” (DG Health, 001–01).

In the views of paediatricians, the absence of follow-up after the referral was also highlighted, especially when the sick child is referred from peripheries to the tertiary care hospitals.

“We don’t have a system of follow-up after referral at all. When a child from a small village is referred to a hospital in the city, there is no follow-up from the referred care facility; whether the child received the required service or not, whether the ambulance was arranged on their own or by the government or by some non-government organization (NGO), we do not have such system right now.” (Paediatrician, 003–01).

While the existence of emergency transportation arrangements for maternal and sick newborn cases in the form of emergency helpline numbers was acknowledged by MNCH managers and planners. However, the non-availability of ambulance service for patients in general and particularly related to sick newborns was cited as a concern.

##### Category 5: absence of a multidisciplinary approach in neonatal case management

Narratives from stakeholders’ interviews also alluded to the lack of coordination mechanism among three different specialties i. e., neonatology, paediatrics, and gynecology in inpatient care units. During emergency events such as fetal distress, the absence of a neonatologist exhibited a lack of coordination among the attending staff, which can risk newborn life.

“Now the protocol in the case of fetal distress is that when an obstetrician is in the operation theater to deliver the baby, there should be a neonatologist present there too. We call them … but they do not reach on time.” (Gynecologist, 006–01).

The underlying reasons that surfaced for the lack of coordination between the specialist cadres were indicative of wrongly perceived role and responsibilities by the respondents;

“A Gynecologist is more interested in maternal health and stillbirths; whereas a paediatrician is only interested in neonatal deaths. There is a lack of coordination between these two cadres.” (MNCH Coordinator, 004–01).

The respondents while sharing their concerns about the lack of coordination between the diverse group of providers rebuked the hospital governance, and strongly emphasized the culture of teamwork while providing care for sick neonates;

“… ..There are communication gaps between the departments … ... It is not only the administration or the government who is responsible. Responsibility, of course, lies upon the gynecologists, obstetricians, and paediatricians as well. We should build an environment of teamwork.” (Gynecologist, 006–01).

##### Category 6: need to institute monitoring system to prevent neonatal deaths and stillbirths

Alongside the above-mentioned issues associated with the poor quality of neonatal care at the facility level, respondents also discussed instituting and strengthening monitoring systems. This includes careful reviews of patient case records, verbal autopsies, perinatology meetings, etc. While highlighting the need for reviewing the patient reports and undertaking verbal autopsies for neonatal deaths and stillbirths, a paediatrician stated;

“Reviewing of reports can give us data, for example, if we report 10 deaths in a month, then we can review what were the causes and check if it was a mistake on our part or from the parents’ side so that we can prevent it from happening again and we can save child’s life.” (Paediatrician, 003–01).

Participants expressed that a mechanism to scrutinize the causes for neonatal deaths is absent at the facility level. In this regard, paediatricians suggested monthly audits by the provincial health department representative and instituting a formal mechanism at the facility and district level to ascertain the causes of neonatal deaths.

“Formal mechanisms with standardized forms can help identify causes and prevent stillbirths and neonatal deaths. If we recommend these forms to the government, then it will get you data about the causes of stillbirths and neonatal deaths.” (Paediatrician, 001–02).

Furthermore, a few of the respondents also highlighted the need for supervisory visits (audits) by the respective provincial health department to ensure correct utilization of the logistic support received from UNICEF at SNCUs.

“Whichever hospital we are working in, whatever equipment we are getting from UNICEF etc. or the training received, there should be a follow-up to see if all of that is provided is being utilized. We don’t have a system for audits.” (Paediatrician, 003–01).

### Theme II: enablers contributing to improved inpatient care for small sick newborns

#### Category 1: improved federal and provincial oversight for reproductive, maternal, and newborn (RMNCH) care

The existence of the RMNCH technical group at the federal and provincial levels was positively viewed by the stakeholders with the potential to improve reproductive, maternal, and newborn care. The respondents viewed that the technical committee is actively working to improve the MNCH across Pakistan. The group comprised of government, non-government, and International Non-Government Organizations (INGOs) with the representation of Health Secretaries, DG (National and Provincial), planning & finance representatives, WHO, alongside UN organizations (WHO and UNICEF). Health care professionals including obstetricians and paediatricians are also reported to be part of the said group. The committee is to promote evidence-based MNCH interventions across the country, alongside advocacy for essential newborn interventions such as umbilical cord care by chlorhexidine at the facility level.

## Discussion

This exploratory study illustrates the compromised quality of care for small and sick newborns at inpatient care units across hospitals in Pakistan. The findings obtained in this study are part of a larger study with a systematic assessment of 23 SNCUs across the country. The findings substantiate concerns expressed by the very stakeholders who are responsible for providing and managing neonatal care of the dismal state of public sector hospitals that is a key contributor to the high neonatal mortality in the country.

Poor quality of facility-based care for the small and sick newborn is a major impediment towards the attainment of universal health coverage and SDG-3. A 12 country study confirmed our results that the interventions with the most perceived bottlenecks are facility-based where rapid emergency care is needed, notably inpatient care of small and sick newborns, treatment of neonatal infections, and KMC [24]. Equally important is to ensure the integration of essential newborn care into countries’ policies and programs [25]. In wake of high neonatal mortality in the country, the availability of essential newborn care standards and guidelines for inpatient care units are essential, which many LMICs lack while offering care to newborns [24, 25]. Similar findings were obtained in our study where participants mentioned about the non-availability of standards and guidelines for neonatal care. The unavailability of neonatal care standards at inpatient care units across the districts reflects that the government’s commitment to improving neonatal health, detailed in MNCH action plans [9] has not translated into actions at the facility level. Furthermore, this important finding also indicates the possibility of communication gap among the health care staff at the inpatient care settings about the availability of existing standards and guidelines.

Studies indicate that countries with high maternal, perinatal, and neonatal mortality have an inadequate and poor quality of health care services and, thus increased emphasis is placed on the need for the standards of care [26]. WHO and UNICEF has a good stock of newborn care standards which can be customized and made available for service providers at the inpatient care units [26–28] in Pakistan.

Ensuring optimum care for small and sick newborns demands adequate infrastructure support with required supplies, equipment, and stock of essential drugs at NICUs. Unfortunately, among the underlying reasons that surfaced for the poor quality of neonatal care were inclusive of inadequate neonatal care infrastructure at DHQs across the country. These findings also correspond with studies in Kenya [29] and Bangladesh [30], exhibiting poor structural support for neonatal care. Health care providers in Bangladesh reported inadequacy of essential logistic support, such as non-functional incubators, alongside the unavailability of the required instruments and reagents to undertake laboratory tests [30]. Our study participants (belonging from non-UNICEF supported facilities) highlighted limited bed capacity across DHQs; resulting in an early discharge of preterm neonates and inadequate infrastructure support in general.

Likewise, findings obtained from the multi-country analysis from five out of 12 countries (from Asian and African regions), also documented limited space for sick newborns and mothers at inpatient care units [7]. Furthermore, poor functionality of neonatal care equipment was also documented from India, with a lack of power backups for SNCUs [31], as was the case in our study. Under essential newborn care, neonatal resuscitation is among the essential component needed for the care of small and sick neonates at inpatient care units. Concerns were raised about the inadequacy of oxygen supplies and equipment at SNCUs. Likewise, findings across LMICs have also demonstrated similar hurdles for neonatal resuscitation, with equipment and supplies such as bags and masks not readily available at the facility level [32].

Furthermore, the stock of a specialized health workforce is essential to ensure quality assured neonatal care services at inpatient care units. Unfortunately, alongside other bottlenecks in neonatal care service delivery at SNCUs, this study also informed about the critical health workforce gap in various cadres; paediatrics, neonatology, and nursing. Unavailability of paediatricians after cesarean section and their delayed arrival has been reported to be associated with wastage of golden hour in this study. The “Golden hour” concept includes practicing all the evidence-based interventions for neonates in the initial sixty minutes for better health outcomes [33]. The shortage of the required health workforce has been perceived as a major obstacle affecting NICUs [34]. Likewise, an inadequate specialized workforce in numbers and skills was also documented from other countries [7].

Our findings also reveal that essential newborn care aspects are not up to the required standards; presumably due to the absence of newborn care standards and poor monitoring at inpatient care facilities. As a result, neonatal deaths are being reported at the facility. This urgently calls for staff training in essential newborn care with a focus on thermal care management. Thermal protection and care of newborn is highly recommended, as it reduces hypothermia; known to contribute to global neonatal mortality and comorbidity of other major causes of neonatal death [35]. The WHO practical guide for the thermal protection of the newborn recommends several key interventions to ensure that the newborn is kept warm. This includes that the place of delivery is warm, newborns are immediately dried and either wrapped or placed on the mother for skin-to-skin contact [36]. Consistent with earlier studies in Nepal [37] and Tanzania [38], our study also reports sub-optimal thermal care management for newborn care by unskilled staff.

Furthermore, a multidisciplinary approach has been highly recommended in neonatal care management, as it leads to integrated care and also facilitates developing rapport with parents [33, 39]. It is not uncommon to note that shortcomings in teamwork for neonatal case management have been reported from earlier studies [40], and our study findings also highlighted the absence of coordination among the care providers from varied disciplines. The underlying reasons could be attributed to the absence of a neonatal care protocol with a mandatory requirement for a multidisciplinary approach in neonatal care, and the lack of accountability mechanism at inpatient care units. Moreover, this study also reaffirmed findings from earlier studies about the inadequate monitoring of sick neonates, with particular attention on instituting a mechanism of perinatal death audits [31, 41]. In this regard, the hospital committee on mortality audit; a concept which is usually non-existent in public sector health care facilities in Pakistan, if gets instituted can prove to be highly beneficial. The committee can review in-hospital mortality data to examine the causes and impact of actions taken to avoid preventable mortality, such as those related to healthcare-associated infections (HAI) [42].

The barriers identified in the study strongly calls to review the accountability mechanism at all three levels; (1) facility level, (2) district health authority, and (3) provincial health departments and clarifying their roles and responsibilities in overcoming the barriers in the provision of care of small and sick newborns. While provincial health departments are responsible to oversee the provision of health care services and liaison with district health offices to provide support to districts. On the other hand, district health offices are mandated to provide needed services in their districts through designated public health care facilities. Facility administration is the third tier with responsibility delegated to deliver and manage health care services. Identification of challenges in inpatient care services to small and sick newborns, therefore, calls for consultative meetings between the facility administrations, district health authorities, supported by the provincial MNCH steering committees to facilitate districts and facility managers develop action plans towards closing the quality gap to improve NYIs care in inpatient care settings. The provincial health department is responsible to resolve financial constraints related to the recruitment of specialized staff at the district level and also to build the capacity of district health authorities across various spheres in delivering optimum care to small and sick newborns at SNCs.

The role of the technical committee on RMNCH was perceived important by the respondents at the federal and provincial levels in terms of improving the quality of inpatient care for small and sick newborns. However, keeping in view the devolved health system in the country, accountability lies with the Provincial Health Departments to strengthen the quality of inpatient care for small and sick newborns in light of the health systems issues discussed above.

The objective of this paper doesn’t aim to draw the comparison of the stakeholder perspectives belonging from UNICEF supported and non-UNICEF supported public health care facilities. The support extended by UNICEF in strengthening public health facilities mainly relates to infrastructural support. In this regard, deficiencies related to infrastructural support were mainly voiced from facilities without UNICEF support.

The study has strengths and limitations. To our knowledge, this is the first study at the national level which has not only captured insights of the frontline care providers deployed at SNCUs, but also explored perspectives of planners and managers involved in the MNCH program on the quality of inpatient care at DHQs in the country. As the scope of the study limits to the public health care facilities, therefore perspectives from the care providers at private care hospitals were not included.

## Conclusions

This qualitative study was insightful in identifying the challenges that influence the quality of inpatient care for small and sick newborns and the resources needed to fix these. Unless these challenges are addressed, neonatal mortality is unlikely to meaningfully decrease in Pakistan soon. The country that has emerged as one of the worst neonatal mortality indicators on the globe essentially needs to strengthen the quality of neonatal health service delivery by providing an enabling environment for improved newborn survival at the facility level. In this regard six actions are recommended at the facility and district level; (1) Strengthening of SNCUs by UNICEF by providing needed equipment and supplies. This can be undertaken by doing gap analysis as part of the facility assessment exercise, (2) Recruitment and deployment of Neonatologists, Neonatal Surgeons, Neonatal Nurses in district hospitals by the District Health Department. For this purpose, District Health Department needs to revise the annual budget to recruit the specialized staff, (3) MNCH steering and coordination committee to liaise with the national and international experts in customizing neonatal care guidelines for inpatient care settings, (4) District Health Department in close collaboration with Provincial Health Department must strengthen pre-service and in-service training programs for health care providers across inpatient care settings in neonatal care management, (5) MNCH steering and coordination committee to conduct monthly audits at inpatient care facilities to supervise staff performance and ensure perinatal and neonatal death reviews. Furthermore, (6) District Health Department must institute a multidisciplinary committee at the hospital level to ensure coordinated involvement of required specialists in offering care to sick neonates**.**

## Data Availability

The datasets used and/or analyzed during the current study are available from the corresponding author on reasonable request.

## Abbreviations

AKU: Aga Khan University
APGAR: Appearance, Pulse, Grimace, Activity, and Respiration
DG: Director General
DHQ: District Headquarter Hospital
ENAP: Every Newborn Action Plan
INGOs: International Non-Government Organizations
LMICs: Low and Middle-Income Countries
MNH: Maternal and Neonatal Health
MNCH: Maternal, Newborn, and Child Health
MS: Medical Superintendent
NICUs: Newborn Intensive Care Units
NGO: Non-government Organization
NYIs: Newborn and Young Infants
NMR: Neonatal Mortality Rate
RMNCH: Reproductive, Maternal and Child Healthcare
SDG: Sustainable Development Goal
SNCUs: Sick Newborn Care Units
TBA: Traditional Birth Attendant
UNICEF: United Nations International for Children’s Education Fund, Country Office, Pakistan
WHO: World Health Organization
